# Effect of soluble amyloid precursor protein-alpha on adult hippocampal neurogenesis in a mouse model of Alzheimer’s disease

**DOI:** 10.1186/s13041-021-00889-1

**Published:** 2022-01-03

**Authors:** Shane M. Ohline, Connie Chan, Lucia Schoderboeck, Hollie E. Wicky, Warren P. Tate, Stephanie M. Hughes, Wickliffe C. Abraham

**Affiliations:** 1 Department of Psychology, Brain Health Research Centre, Brain Research New Zealand, University of Otago, Dunedin, New Zealand; 2 Department of Physiology, Brain Health Research Centre, Brain Research New Zealand, University of Otago, Dunedin, New Zealand; 3 Department of Biochemistry, Brain Health Research Centre, Brain Research New Zealand, University of Otago, Dunedin, New Zealand

**Keywords:** Alzheimer’s disease, Adult neurogenesis, sAPPα, Dentate gyrus, APP/PS1, Astrocytes, Proliferation, Differentiation

## Abstract

Soluble amyloid precursor protein-alpha (sAPPα) is a regulator of neuronal and memory mechanisms, while also having neurogenic and neuroprotective effects in the brain. As adult hippocampal neurogenesis is impaired in Alzheimer’s disease, we tested the hypothesis that sAPPα delivery would rescue adult hippocampal neurogenesis in an APP/PS1 mouse model of Alzheimer’s disease. An adeno-associated virus-9 (AAV9) encoding murine sAPPα was injected into the hippocampus of 8-month-old wild-type and APP/PS1 mice, and later two different thymidine analogues (XdU) were systemically injected to label adult-born cells at different time points after viral transduction. The proliferation of adult-born cells, cell survival after eight weeks, and cell differentiation into either neurons or astrocytes was studied. Proliferation was impaired in APP/PS1 mice but was restored to wild-type levels by viral expression of sAPPα. In contrast, sAPPα overexpression failed to rescue the survival of XdU^+^-labelled cells that was impaired in APP/PS1 mice, although it did cause a significant increase in the area density of astrocytes in the granule cell layer across both genotypes. Finally, viral expression of sAPPα reduced amyloid-beta plaque load in APP/PS1 mice in the dentate gyrus and somatosensory cortex. These data add further evidence that increased levels of sAPPα could be therapeutic for the cognitive decline in AD, in part through restoration of the proliferation of neural progenitor cells in adults.

**Supplementary Information:**

The online version contains supplementary material available at 10.1186/s13041-021-00889-1.

## Introduction

Alzheimer’s disease (AD) is an aging-related neurodegenerative disorder that is the most common form of dementia. It is characterised by a build-up of toxic soluble oligomers of the amyloid-β (Aβ) peptide (both intra- and extracellularly), aggregation of the Aβ into extracellular insoluble plaques, hyper-phosphorylation of tau proteins with associated generation of toxic neurofibrillary tangles, and impaired cerebrovasculature [1]. These changes trigger neuroinflammation, synapse loss, impaired neurogenesis and ultimately cell death. Despite the intense international research efforts addressing AD, the development of effective therapies has been painfully slow. Accordingly, new therapeutic approaches, based on an increased understanding of the disease mechanism as well as the mechanisms of action of possible therapeutic molecules, are urgently required. A commonly held view regarding the primary or at least major pathophysiology in AD centres on the amyloid cascade hypothesis [2]. This states that the over-accumulation of Aβ, either by enhanced cleavage of the parent protein amyloid precursor protein (APP), or by decreased clearance, is a critical step in developing AD. More recently, the ‘beta amyloid dysfunction hypothesis’ has been proposed whereby it is the formation of misfolded Aβ soluble synaptotoxic aggregates that is responsible for the pathology, and their formation also leads to a depletion of the functionally important Aβ monomer [3].

Full-length amyloid precursor protein (APP) is a ubiquitously expressed protein that has been localised to synaptic membranes, including both the postsynaptic density and adhesion patches, suggesting that neuronal APP plays a role in both signal transduction and cell adhesion [4]. Proteolytic processing of APP in vivo yields protein fragments which themselves regulate neuronal function [5]. The γ/β secretases release the Aβ fragment and the N-terminal fragment sAPPβ, whereas α-secretase cleavage within the Aβ peptide sequence prevents its production and releases the larger N-terminal fragment soluble amyloid precursor protein-alpha (sAPPα). sAPPα differs from sAPPβ by only a 16 amino acid extension at its C-terminus, but is generally 100-fold more potent in regulating neuronal function [6].

Exogenously administered sAPPα is both neuroprotective and neurotrophic [7] as evidenced, for example, through promoting survival of cultured neurons [8], facilitating neurite outgrowth and reducing neural damage after diffuse traumatic brain injury in rats [9]. Exogenous sAPPα improves spatial memory processes [10], and visual discrimination performance and memory retention in mice [11], while cerebrospinal fluid levels of sAPPα correlate with spatial memory abilities in rats [12] and in people with Alzheimer’s disease [13]. Together these findings establish sAPPα as a regulator of neuronal and particularly memory mechanisms that may be disrupted in AD. In our studies, we have confirmed the neuroprotective actions of sAPPα, and have also shown that acute administration of sAPPα increases synaptic protein synthesis [14], the expression of neuroprotective genes [15], the function of N-methyl-D-aspartate receptors and the induction of long-term potentiation [16]. We have also shown that expression of human sAPPα, when generated by delivery of a lentivirus directly to the hippocampus in vivo, fully rescued spatial memory in the water maze and caused a partial rescue of LTP in CA1 in vitro, but without affecting plaque burden [17]. Similarly, Fol et al. found that expression of mouse sAPPα via adeno-associated virus 9-mediated gene transfer into APP/PS1 hippocampus rescued spatial memory, LTP and spine density, while also partially reducing the plaque load [18].

It is with great interest that historical findings of neurogenesis in the adult brain [19] have been replicated and extended over the past 50 years [20–23]. Harnessing the adult brain’s neurogenic potential has been proposed to have potential for promoting recovery from a variety of brain disorders characterised by neuronal loss. However, there is now considerable evidence that adult neurogenesis has a normal and important role in cognition. There are two primary neurogenic regions in the adult brain, the subventricular zone (SVZ) lining the lateral ventricle, and the subgranular zone (SGZ) in the dentate gyrus region of the hippocampus. The dentate gyrus contributes to the processing of spatial information in the hippocampus, particularly with regards to discrimination of spatial patterns [24–26]. Intriguingly, neurons recently born in adult animals, once differentiated and connected within the dentate network, show enhanced LTP [27] and preferential incorporation into the neuronal networks storing new information [28]. In addition, conditions that reduce dentate neurogenesis, such as stress, aging or disease [29–31], also lead to deficits in spatial memory. Adult neurogenesis is impaired in mouse models of familial AD (e.g., APP_swe_/PS1_ΔE9_; APP/PS1), [32–34] and in humans with AD [23]. Based on these findings, it has been proposed that continued generation of dentate neurons is essential for the ongoing ability to perform pattern separation of spatial and other types of information, thereby facilitating memory storage elsewhere in the hippocampus [5, 35, 36]. Thus, recovery of spatial cognitive function in disease may require treatments that enhance ongoing dentate neurogenesis.

With the above considerations in mind, we sought to increase adult neurogenesis by overexpression of sAPPα in the dentate gyrus of APP/PS1 mice. We used adeno-associated virus 9-mediated gene transfer with a mouse sAPPα construct similar to that used in Fol et al. [18] to transduce cells in the dentate gyrus of wild-type (WT) and APP/PS1 mice in order to increase neurogenesis in vivo. We found that cell proliferation was decreased in the APP/PS1 mice, but this was rescued by the overexpression of sAPPα. Survival of adult born cells, including neurons and astrocytes, was impaired in APP/PS1 mice, however sAPPα overexpression did not rescue this deficit. We found that sAPPα overexpression increased astrocytic, but not neuronal survival in the granule cell layer (GCL) of APP/PS1 mice. sAPPα overexpression increased astrocytic differentiation, as indicated by the percentage of adult-born astrocytes in the GCL irrespective of genotype. In contrast, neuronal differentiation was mainly unchanged in the GCL of both genotypes. sAPPα overexpression also reduced β-amyloid plaque burden in the dentate gyrus (DG) and cortex.

## Methods

### Animals

All animal-use procedures were approved by the University of Otago Animal Ethics Committee and conducted in accordance with New Zealand Animal Welfare legislation. Adult female APP/PS1 and wild-type C57BL/6 J mice were used (average age 8 months at time of virus injection). The APP/PS1 mice on a C57BL/6J-congenic background harboured mutations in human APP_695_ (the Swedish mutations: K670N, M671L) and human PS1 exon nine deletion (PS1_ΔE9_). Genotyping was carried out by tail tip biopsy with a PCR reaction. Mice were individually housed (post-virus injection only) at 21 °C and maintained on a 12 h light/dark cycle (lights on 6 am). All animals had access to food and water ad libitum.

### Viral vectors and administration

The viral vector encoding AAV9-syn-HA-HA-sAPPα (mouse sequence from APP_695_, experimental) was commercially obtained from the University of Pennsylvania (Philadelphia, USA). The AAV9-syn-EGFP (control) vector was packaged in-house through the Otago Viral Vector Facility, Mārama platform of Brain Research New Zealand. Both viruses used the minimal human synapsin 1 promoter (syn). The plasmid containing the syn-HA-HA-sAPPα coding sequence (codon optimised for mouse) was designed locally and generated by GeneArt (Thermo Fisher) before packaging into AVV9 particles at the University of Pennsylvania. A similar (AAVp-syn-HA-HA-sAPPα) virus has been successful in producing sustained sAPPα overexpression in APP/PS1 mice [18]. The AAV9-syn-HA-HA-sAPPα was delivered at a titre of 1 × 10^14^ GC/mL, and the control vector (AAV9-syn-EGFP) was administered at 1 × 10^12^ GC/mL.

Mice (23 female, 8 months old) were anaesthetised by inhalation of 2% isoflurane in oxygen and were placed in a stereotaxic frame (Kopf, CA, USA). A small hole was drilled directly above the injection site. A 33-gauge bevelled syringe needle (WPI, Florida, USA) was lowered to the dorsal dentate gyrus of the hippocampus with coordinates of (in mm from bregma) AP -2, ML ± 1.4 and DV -1.8. Mice received bilateral 1 µL injections of either AAV9-HA-HA-sAPPα or the AAV9-EGFP virus into the DG at a rate of 0.15 µL /min controlled by an infusion micropump (Kopf, CA, USA). At the end of the injection, the needle was left in situ for a further 5 min to allow diffusion before slow withdrawal. After injection, the incision was sutured and anaesthesia was reversed by removing from isoflurane. In total, 7 WT and 5 APP/PS1 mice were injected with the sAPPα virus, while 6 WT and 5 APP/PS1 mice were injected with the EGFP control virus.

### Thymidine analogue injections

**Fig. 1:**
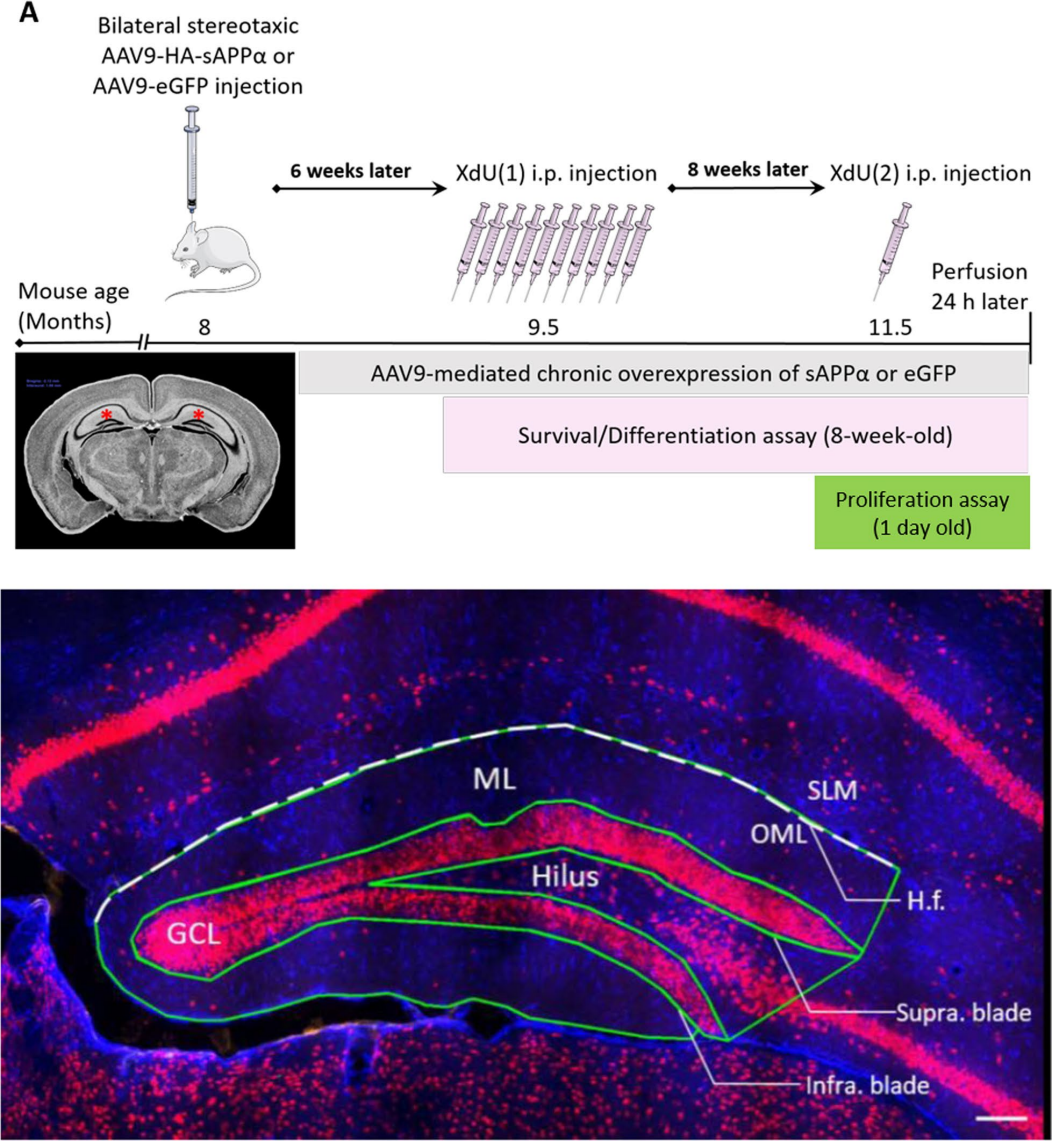
Injection paradigms and definition of subregions of the dentate gyrus. **A, **upper panel, intrahippocampal injection paradigm for the experimental or control virus at the 8-month animal age, with all animals killed at 11.5 months of age. XdU (CldU or IdU) injections 6 weeks later were used for survival and differentiation studies, while the other XdU was injected 14 weeks post-injection for the proliferation study. **B,** lower panel, regions of the dentate gyrus were as outlined: *ML* molecular layer, *H.f.* hippocampal fissure, *GCL* granule cell layer, *OML* outer molecular layer, *SLM* stratum lacunosum moleculare, *Supra blade* suprapyramidal blade, *Infra blade* infrapyramidal blade. Scale bar: 100 µm

The thymidine analogues, 5-chloro-2’-deoxyuridine (CldU) and 5-iodo-2’-deoxyuridine (IdU), collectively termed XdU, were used to birth-date adult born cells. Mice were injected i.p. with CldU (#105478, MP Biomedicals, Ohio, USA) and IdU (#100357; MP Biomedicals, Ohio, USA) at two time points using a 27-gauge needle. Six weeks following AAV9 delivery, 9.5 mo old mice received i.p. injections on one XdU (50 mg/kg BrdU equivalent in 0.9% saline) twice a day at eight hour intervals for five days (10 total injections). Eight weeks after the last XdU injection (15 weeks after virus delivery), 11.5 mo old mice received a single i,p. injection of the other XdU (200 mg/kg BrdU equivalent). Mice were then perfused one day later. This allowed for the effect of sAPPα treatment to discriminate between a population of newly proliferated cells (1 day old) and a population of mature adult-born cells (8 weeks old), the latter allowing assessment of cell survival and differentiation (Fig. 1). The protocols followed the procedures outlined in Ohline et al. [37].

In a separate preliminary experiment to assess XdU antibody specificity, two 2 months old C57BL/6 J mice were injected with 200 mg/kg (i.p.) BrdU equivalent of both CldU (171 mg/kg, 5 weeks before perfusion) and IdU (230 mg/kg, 2 weeks before perfusion). These animals were studied using the procedures below for antibody specificity. The virus-injected mice were used for analysis of (1) proliferation of adult-born cells or (2) survival and differentiation of adult-born cells.

### Immunofluorescence procedures for adult-born cell analysis

All animals were deeply anaesthetised by an i.p. injection of pentobarbital (30 mg/mL, 150 µl per mouse). The animals were transcardially perfused with 20 mL of phosphate buffered saline (PBS, 0.01 M PB, 0.9% (w/v) NaCl) followed by 20 mL of ice-cold 4% (v/v) paraformaldehyde (PFA) in phosphate buffer. The brain was removed and post-fixed in 4% PFA at 4 °C overnight and subsequently transferred to a 30% (w/v) sucrose in 0.1 M PB solution for a minimum of 48 h for cryoprotection. Brains were then sectioned at 40 µm thickness using a cryostat (Leica Biosystems, Mannheim, Germany). Free-floating sections were kept using a cryoprotectant solution containing ethylene glycol (30% v/v), sucrose (30% w/v) and phosphate buffer (0.1 M) at − 20 °C until used for immunofluorescence imaging.

Every sixth section through the entire hippocampus (approximately 8 slices per animal) was subjected to immunofluorescence imaging and analysis as follows. Eight sections were used for the proliferation study (second XdU/NeuN), while another eight sections were used for the survival and differentiation study (first XdU/NeuN/GFAP). Slices were washed overnight at room temperature in Tris-buffered saline (TBS) to remove any residual cryoprotectant solution. In all cases, the slices were incubated in 2 M HCl for 1 h at 37 °C. Subsequently, boric acid (0.1 M, pH 8.5) neutralised this solution (10 min). After washing with TBS sections were blocked using a TBS containing 3% normal goat serum and 0.1% Trion-X-100 for 1 h at room temperature. Sections were incubated for 48 h at 4 °C shaking in the primary antibodies of interest, washed in the TBS Triton-X solution and then incubated for four hours with shaking in the appropriate fluorophore-conjugated secondary antibodies diluted in the blocking solution at room temperature. Slices were washed and mounted on gelatin-coated microscope slides using an anti-fade mounting solution containing glycerol (80% v/v) with 1,4-phenylenediamine dihydrochloride in phosphate buffer (0.1 M). Primary antibodies were: BrdU/CldU (rat, 1:250, Abcam ab6326), BrdU/IdU (mouse, 1:250, BDSciences 347580), NeuN (guinea pig, 1:500, Synaptic Systems, 266-004), GFAP (rabbit, 1:1000, DAKO Z033401-2), 6E10 (mouse, 1:1000, BioLegend, 803002), HA-tag (mouse, 1:1000, BioLegend 901514). The secondary antibodies, all conjugated with AlexaFluor (AF) were raised in goat, were purchased from Invitrogen and had the following catalogue numbers: anti-rat (AF488, 1:500, A11006); anti-mouse (AF555, 1:500, A21414); anti-guinea pig, AF647 (1:250, A21450); anti-rabbit, (AF405, 1:500, A31556); anti-rat (AF555, 1:500, A21434).

### Image analysis

Digital images of the hippocampus were captured using a Nikon Ni-E confocal microscope equipped with a Nikon C2plus camera using a 40 × objective (Plan Apo: N/A = 0.95; Nikon Instruments, Tokyo, Japan). Z-stacks of the DG from both hemispheres were generated using NIS-Elements, Advanced Research 4.50 software. Four excitation wavelengths were used: 405, 488, 555 and 647 nm (pinhole 1.2 AU). Using 1:6 sections throughout the hippocampus, 14–16 images (from 7–8 sections) were acquired using mosaic and z-stack imaging technique for each analysis. A total of 5 z-slices at 8 µm intervals through the 40 µm section were acquired. Care was taken to identify each cell in a single optical slice, so that it was not double-counted if it appeared in a neighbouring slice. For the antibody specificity test, across 8 slices per animal (two animals total), the number of cells labelled with rat anti-BrdU (CldU^+^), cells labelled with mouse anti-BrdU (IdU^+^) and cells double-labelled with rat anti-BrdU and mouse anti-BrdU (CldU^+^/IdU^+^) were counted in the GCL. The percentage of CldU^+^/IdU^+^ cells in the GCL was calculated per animal. For the proliferation assay, 14–16 z-stacks of the entire DG were analysed per mouse using Fiji/ImageJ version 1.5.3 (NIH, USA). Each XdU^+^ cell in the SGZ was counted in optical slices from individual z-stacks. NeuN immunostaining was used to visualise the GCL and define the SGZ, which was set as 20 µm width (approximately two granule cell widths) on either side of the margin between the GCL and hilus. A maximum intensity-projection image was rendered for each DG from the z-stacks and the length of the entire DG was measured in ImageJ to give a cell linear density of XdU^+^ cells/mm length of DG. In a separate set of 1:6 sections through the DG for each animal, in the cell survival assay, eight-week-old XdU^+^ cells throughout the DG, including the molecular layer (ML), the hilus and the GCL were counted in each subregion. NeuN immunostaining was used to identify the subregions. Maximum projection images of each DG were taken and the area (in mm^2^) defining each subregion was measured in ImageJ. XdU^+^ counts per mm^2^ were taken for each region as an indication of cell survival. For the differentiation assay in the same sections used for the cell survival assay, nuclear XdU staining was first confirmed before each XdU^+^ cell was individually inspected for colocalisation with NeuN for neuronal phenotype (NeuN^+^/XdU^+^) or with GFAP (GFAP^+^/XdU^+^) for astrocytic phenotype or with neither NeuN nor GFAP (NeuN^−^/GFAP^−^/XdU^+^) in optical slices from individual z-stacks. Cell counts were performed separately in three subregions of the DG: the GCL, the ML and the hilus for both hemispheres. For a cell to be considered NeuN^+^/XdU^+^, XdU^+^ staining had to localise within the dentate NeuN^+^ cell. Due to the localisation of XdU in the nucleus only XdU^+^ cells that co-localised with GFAP^+^ soma, but not processes across two colours were counted as double-labelled. NIS-Elements was used in tandem with ImageJ to scan stacks of magnified single cells in the z-plane to avoid duplicate counts. All quantitative assessments were performed blinded to the experimental groups.

### Determination of virus-mediated expression

To identify sAPPα or HA expression, brain sections (1:6 through the hippocampus) were washed in phosphate buffer (PB 0.1 M), then incubated in blocking solutions (PB containing 10% (v/v) normal goat serum with 0.1% (v/v) Triton X-100). Sections were incubated overnight with shaking at 4 °C with primary antibodies. The following day, sections were washed with PB, incubated with shaking for 2 h with shaking with secondary antibody at room temperature. Cell nuclei were counterstained with 4’,6’-diamino-2-phenylindole (DAPI, Life Technologies, NZ) contained in the anti-fade mounting medium as above before adding coverslips. Sections treated with only the secondary antibody were used as a control. To examine the spread of sAPPα overexpression throughout the dorsal–ventral extent of the hippocampus, sections from sAPPα-treated mice were immunolabelled with mouse anti-6E10 (sAPPα) and DAPI or mouse anti-HA and DAPI. Subsequently, the spread was visualised using Cytation 5 (Biotek, Vermont, USA) and a 4 × objective. An image of the whole slide, containing six to eight sections per mouse was scanned and captured using Gen5 Image + version 2.09 software.

### Congo red staining

Slides were prepared with eight sections per APP/PS1 mouse (1:6 through the hippocampus) and Congo red was used to stain the sections to reveal amyloid plaques, with nuclei labelled with DAPI. Congo red staining and DAPI were visualised on a Nikon Eclipse Ti2 fluorescence microscope. Images of Congo red and DAPI were captured using a Nikon DC Qi2 camera, a 10 × objective (Plan Apo; N/A = 0.30; Nikon Instruments, Tokyo, Japan) and NIS-Element F 4.6 software. Images were converted to 8 bit, a threshold value was determined and maintained for all images, and the percentage area covered by plaques was calculated using the ImageJ algorithm. As sAPPα expression was observed in the cortex overlying the hippocampus, the cortex and CA1 as well as the DG region were all analysed for plaque burden.

### Statistics

All statistical analysis was performed using GraphPad Prism v8.4.3. In all cases, P-values < 0.05 were considered significant. One animal was excluded from the Tg-control group on the basis of a Grubbs’ outlier test (Alpha = 0.05), as the animal displayed an abnormally high density of newly proliferated cells. Data were expressed as mean values ± standard error of the mean (SEM). For the analysis of the linear or area density or percentage of adult-born cells, differences between groups determined using ANOVA test followed by Tukey’s post-hoc test when significant main effects or interactions were detected. Dorsal and ventral data were collected from all mice; thus, a two-way repeated measure ANOVA was used to determine whether there were regional differences in adult hippocampal neurogenesis (AHN) across the longitudinal extent of the hippocampus between groups. Subsequent multiple comparisons were performed using a Sidak’s post hoc test when appropriate. A two-tailed unpaired Student’s *t*-test was used to compare the means of the Congo red plaque area analysis.

## Results

### Antibody specificity

The experimental design of the present study involved the administration of CldU and IdU at separate times to birthdate two distinct cohorts of adult-born cells within the same animal. The presence of both CldU and IdU, however, requires anti-BrdU antibodies that bind specifically to the target antigen of the injected CldU and IdU, respectively, to avoid cross-reactivity that produces non-specific staining. Therefore, we validated the specificity of the antibodies to injected CldU and IdU, respectively. The antibodies employed in this study, mouse anti-BrdU and rat anti-BrdU, were demonstrated previously to be selective for IdU and CldU, respectively [37]. However, the manufacturer of these antibodies had changed, and thus we tested the specificity of the antibodies used for this particular study.

**Fig. 2:**
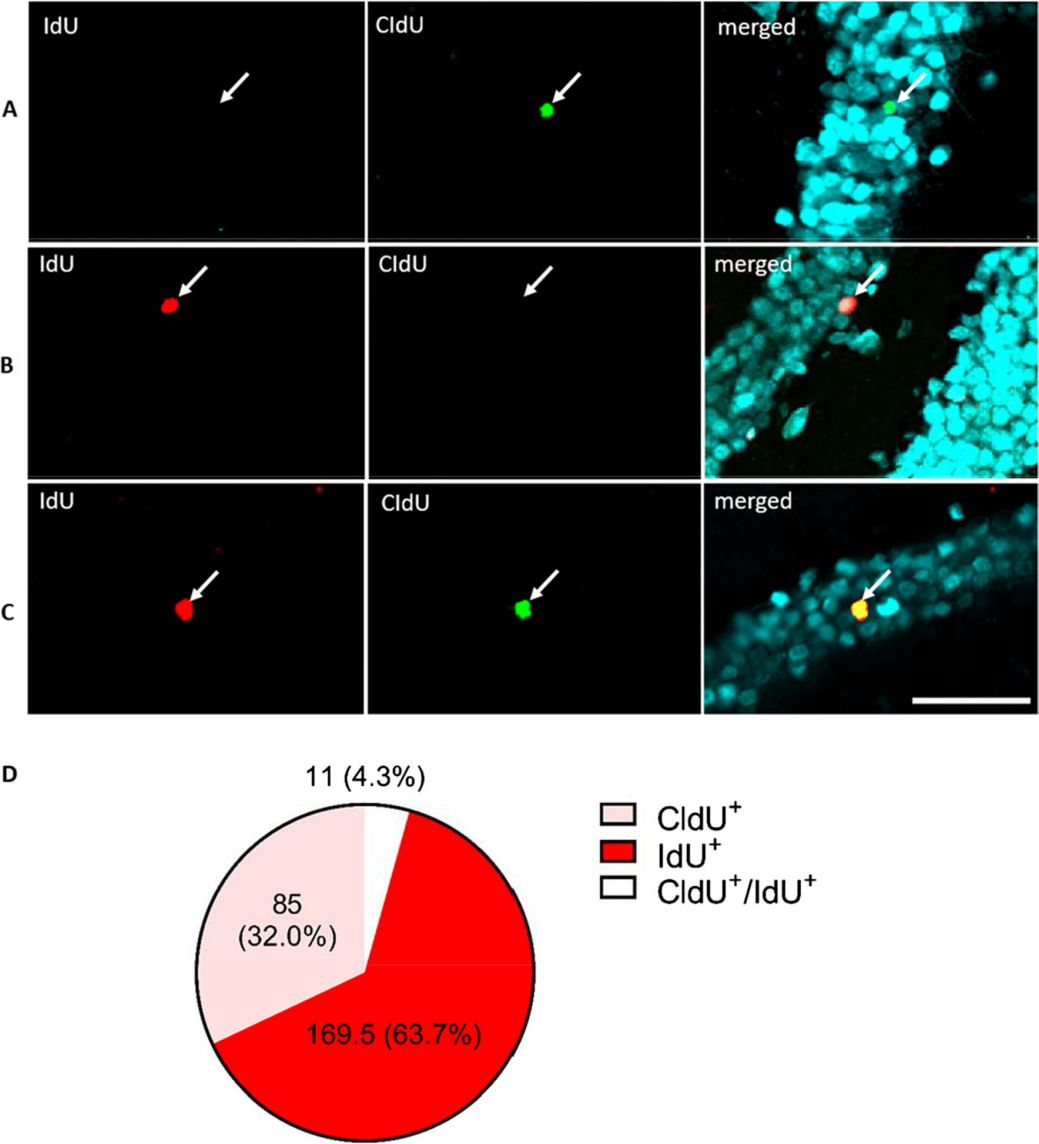
Test of CldU and IdU antibody specificity. **A** Cell in the GCL (NeuN, cyan) was positive for only rat anti-BrdU (CldU, green) antibody. **B** Cell in the GCL (NeuN, cyan) was positive for only mouse anti-BrdU (IdU, red) antibody. **C** Cell in the GCL (NeuN, cyan) was positive for both mouse anti-BrdU (IdU, red) and rat anti-BrdU (CldU, green) antibodies, indicating a non-specific reaction (yellow). Scale bar: 25 µm. **D** The mean number of CldU + cells (pink), IdU + cells (red) and CldU + /IdU + cells (white). 4.3% denotes the mean percentage overlap of IdU and CldU co-labelled cells

In the WT mice injected with both CldU and IdU, both i.p., at 200 mg/kg BrdU equivalent at two different time points (CldU at 5 wk and IdU at 2 wk before perfusion), only 4.3% of cells were double-labelled for CldU and IdU (Fig. 2D). Thus, the antibodies used to detect the injected CldU and IdU were highly specific (95.6%) to CldU and IdU, consistent with our previously published result for the antibodies from the original manufacturer (97% accuracy, [37]). We note that IdU gave a higher percentage of positive cells, indicative of the reduced cell death at the closer time-point (2 weeks before analysis).

### Confirmation of virus-mediated expression of sAPPα

**Fig. 3:**
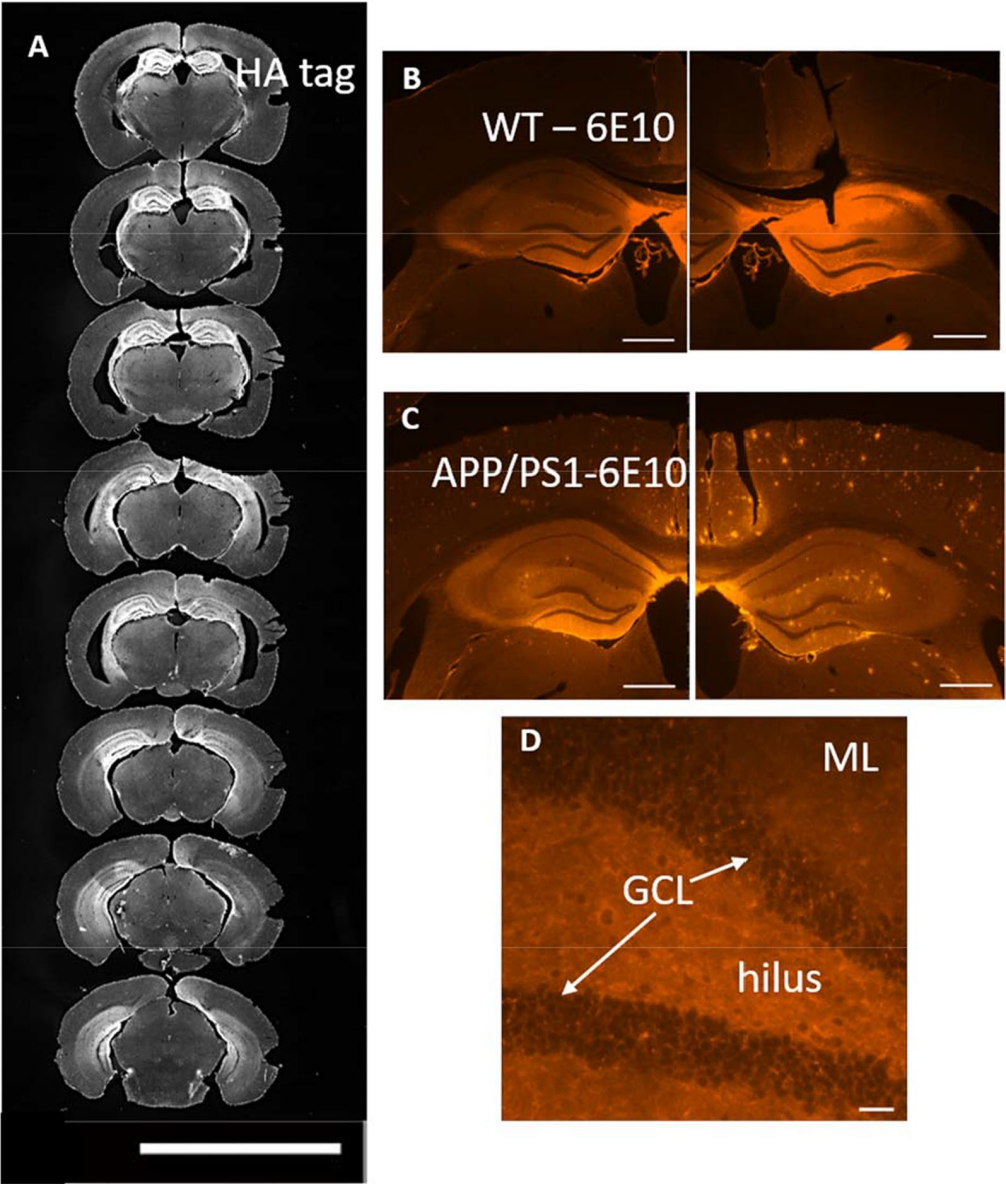
Extent of HA and sAPPα expression throughout the dentate gyrus*.* **A** Representative expression of HA through the dorsal–ventral extent of the hippocampus of a virally transduced mouse. Scale bar: 5 mm. **B** Representative expression of sAPPα in a wild-type mouse hippocampus labelled with the 6E10 antibody. **C** Representative expression of sAPPα in an APP/PS1 mouse hippocampus also labelled with the 6E10 antibody. Note that this antibody directed against the first half of Aβ detects sAPPα but also labels amyloid-beta plaques. Scale bar: 1 mm. **D** A 40 × image of the sAPPα expression in the granule cell layer of a wild-type mouse. sAPPα is seen in cell bodies and, as expected, diffusely in the neuropil as sAPPα is expected to be secreted from the cells into the extracellular space. Scale bar: 25 µm

Adeno-associated viral vectors (AAV9) encoding HA-tagged human sAPPα (from APP695) or control green fluorescent protein (GFP), both under the control of the neuron-specific synapsin promoter, were stereotaxically injected into the dorsal DG. This direct targeting to the dorsal DG, with sAPPα secreted from the expressing cells, exposed even likely non-expressing neural progenitor cells to a sAPPα-rich environment, potentially optimising the regulation of adult hippocampal neurogenesis. Mice were housed for six weeks to ensure maximum transgene expression before the XdU mitotic marker administration to assay for adult-born cell survival/differentiation. Mice exhibited no abnormal behaviour or death during the experiment, suggesting that the sustained overexpression of sAPPα or EGFP was well tolerated. Immunofluorescence was performed to qualitatively assess the spread of virus-mediated expression of sAPPα (indicated by 6E10, an antibody that binds to sAPPα directly) and of HA (the tag fused to sAPPα) in hippocampal brain sections of AAV-HA-sAPPα injected mice (Fig. 3). Successful viral transduction was seen in all animals. HA expression was generally seen throughout the dorsal to ventral extent of the hippocampus (Fig. 3A), as was sAPPα expression (representative sections, Fig. 3B–D). In nearly all cases the spread of the viral transduction occurred through the entire hippocampus. At a minimum, the HA and sAPPα expression was found only in the dorsal hippocampus.

### Cell proliferation in the SGZ

To determine the effect of genotype and sAPPα expression on cell proliferation, the linear density (cells/mm) of 1-day-old XdU^+^ cells was quantified in the SGZ. The nuclei of newly proliferated XdU^+^ cells had similar morphology—small, irregular, and occasionally appearing in punctate clusters in both WT and Tg mice.

**Fig. 4:**
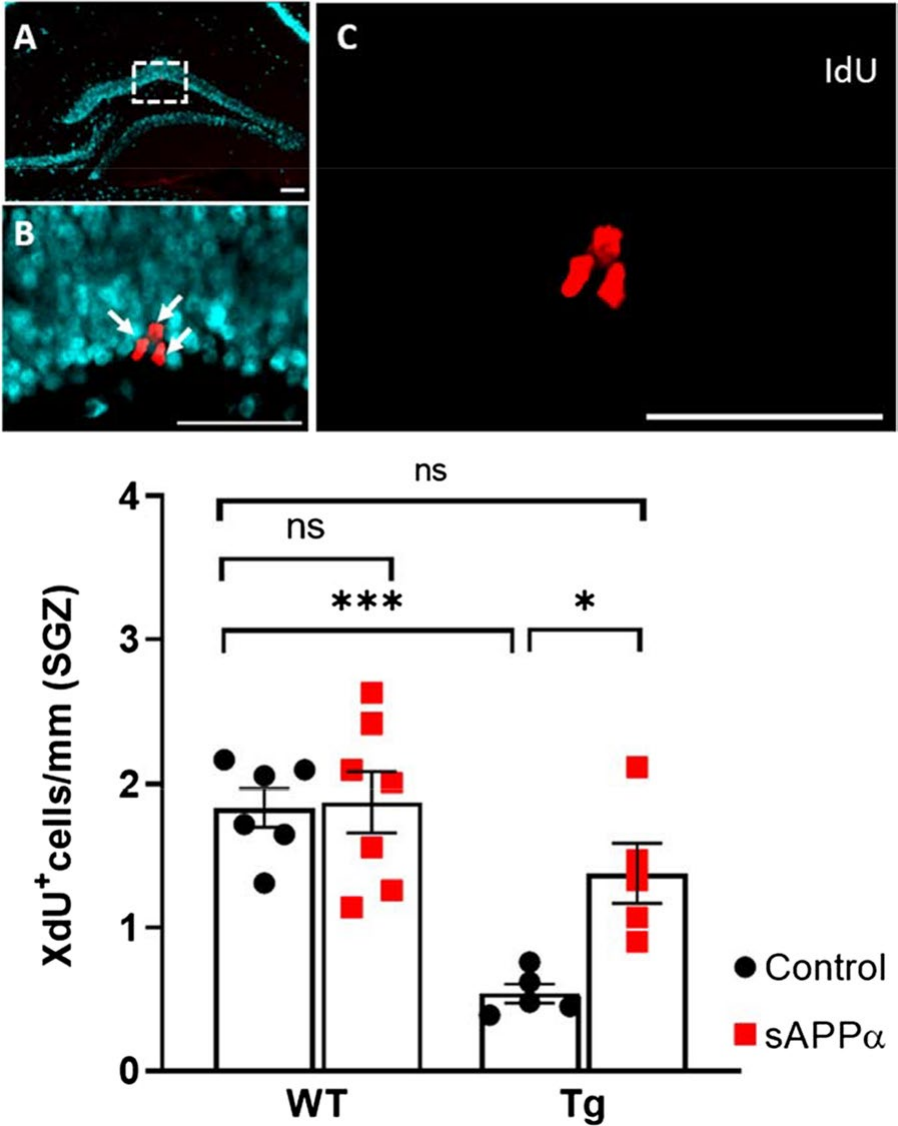
sAPPα effects on cell proliferation in the subgranular zone (SGZ)*.* **A** NeuN (cyan) labelled neurons in the dentate granule cell layer. Scale bar: 100 µm. **B** Insert from **A** showing proliferating cells (IdU, red) in the SGZ. Scale bar: 50 µm. **C** Cluster of proliferating cells (red) in the SGZ labelled by IdU, as indicated by arrows in (**B**). Scale bar: 50 µm. Bottom panel Results from sAPPα overexpression on proliferation in wild-type (WT) and Tg animals (WT-control vs. Tg-control: 1.84 ± 0.14 vs. 0.54 ± 0.07 cells/mm). ns, not significant; *, p < 0.05; ***, p < 0.001 assessed by two-way ANOVA followed by Tukey’s post-hoc test. Data expressed as mean ± SEM

A two-way ANOVA revealed a significant main effect of genotype (F_(1, 19)_ = 24.67, *p* < 0.001), indicating that Tg mice had a reduced linear density of XdU^+^ cells relative to WT mice. Post-hoc analyses revealed that this overall effect was due to Tg-control mice having a significantly reduced linear density of XdU^+^ cells by ~ 71% in the SGZ compared to WT-control mice (WT-control vs. Tg-control: 1.84 ± 0.14 vs. 0.54 ± 0.07 cells/mm, *t*_(19)_ = 7.063, *p* < 0.001; Fig. 4).

AAV-HA-sAPPα-treated mice had a significantly increased linear density of XdU^+^ cells compared with AAV-GFP control mice (main effect of sAPPα; F_(1, 19)_ = 5.920, *p* = 0.025). Importantly, post-hoc analyses revealed that this overall effect was due to Tg-sAPPα mice having a significantly greater linear density of XdU^+^ cells by ~ 150% in the SGZ compared to Tg-control mice (Tg-control vs*.* Tg-sAPPα: 0.54 ± 0.07 vs. 1.38 ± 0.21 cells/mm, *t*_(19)_ = 4.377, *p* = 0.028; Fig. 4). In addition, the linear density of XdU^+^ cells in the SGZ was returned to close to the WT-control level (WT-control vs*.* Tg-sAPPα: 1.84 ± 0.65 vs. 1.38 ± 0.21 cells/mm, *t*_(19)_ = 2.492, *p* = 0.321). However, there was no effect of sAPPα on the proliferation of cells in the WT animals (post-hoc analyses: WT-control vs*.* WT-sAPPα: 1.84 ± 0.65 vs. 1.88 ± 0.25 cells/mm, *p* = 0.998). Thus, virus-mediated sAPPα expression rescued the cell proliferation deficit in the SGZ of Tg-control mice, without affecting proliferation in the WT mice.

### Survival of adult-born cells in the DG

**Fig. 5:**
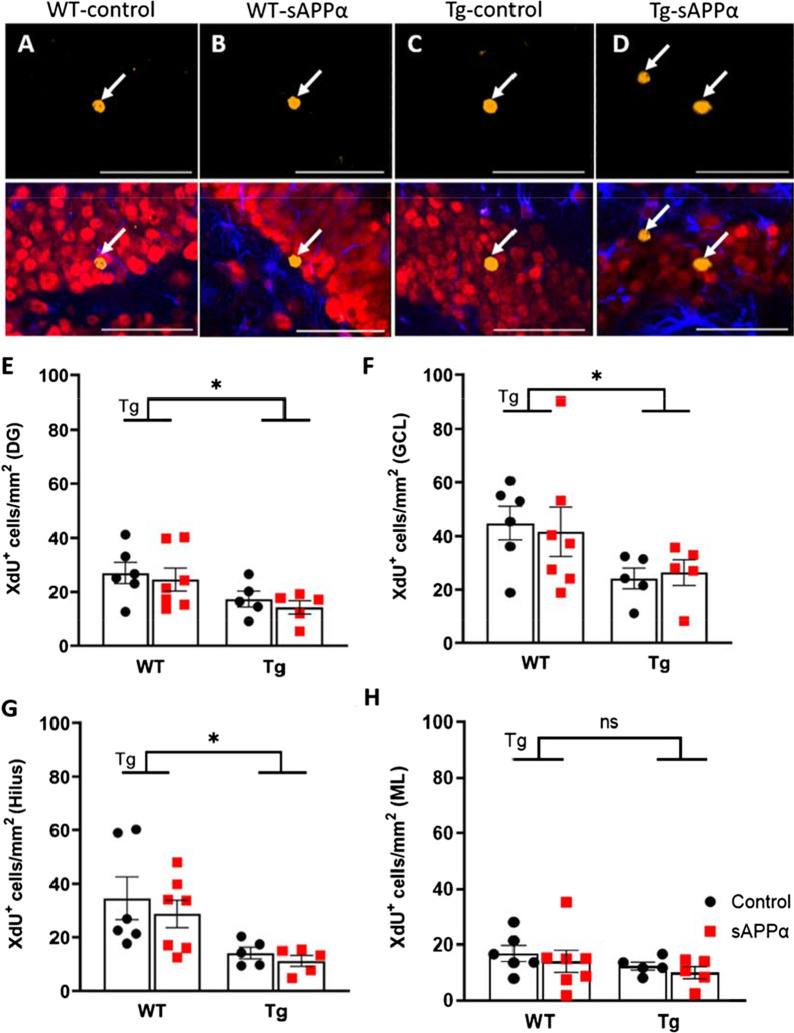
sAPPα effects on cell survival in the DG*.* **A**–**D** top Representative optical sections of CldU immunostaining (yellow, arrows) from control (**A** and **C**) and sAPPα-treated mice (**B** and **D**). **A**–**D** bottom Confocal images triple-labelled with CldU (yellow, arrows), NeuN (red, neurons) and GFAP (blue, astrocytes) in the adult DG. Scale bars: 50 µm. Results of the survival assay (area density of XdU^+^ cells) in (**E**) the whole dentate gyrus, (**F**) the granule cell layer, (**G**) the hilus, and (**H**) the molecular layer. Tg, main effect of genotype; sAPPα, main effect of sAPPα treatment as assessed by two-way ANOVA. *p < 0.05. Data expressed as mean ± SEM

To determine the effect of genotype and sAPPα on cell survival, the area density (cells/mm^2^) of 8-week-old adult-born XdU^+^ cells was quantified in the GCL, ML, and hilus. Cell counts were performed in each DG subregion separately and also combined to determine adult-born cell survival in the whole DG. Immunofluorescence against XdU revealed mature XdU^+^ nuclei in all groups (Fig. 5A–D).

### Effect of APP/PS1 genotype

In the whole DG, there was a significantly reduced density of XdU^+^ cells in Tg mice compared to WT mice (main effect of genotype F_(1, 19)_ = 7.034, *p* = 0.016; Fig. 5E). This effect was due principally to significantly reduced XdU^+^ densities in the GCL and hilus (GCL (F_(1, 19)_ = 6.330, *p* = 0.021); hilus (F_(1, 19)_ = 11.96, *p* = 0.003; Fig. 5F,G), with no significant genotype main effect for the ML (Fig. 5H).

### Effect of sAPPα overexpression

No main effects of sAPPα treatment were detected on XdU^+^ cell area density in the GCL (F_(1, 19)_ = 0.004, *p* = 0.950), ML, (F_(1, 19)_ = 0.6774, *p* = 0.421), hilus (F_(1, 19)_ = 0.6484, *p* = 0.431), or the whole DG (F_(1, 19)_ = 0.534, *p* = 0.474). Moreover, there was no genotype × sAPPα treatment interaction when measured at the level of the GCL only (F_(1, 19)_ = 0.146, *p* = 0.707) or the whole DG (F_(1, 19)_ = 0.008, *p* = 0.930). These results indicate that sAPPα overexpression did not affect the survival of adult-born cells across the 8-week survival period.

### Survival of adult-born neurons and astrocytes

We also assessed the effect of genotype and sAPPα on the survival of specifically adult-born neurons and astrocytes by quantifying the area density of eight-week-old adult-born XdU^+^ cells co-expressing NeuN or GFAP in the GCL and the whole DG, which included the GCL, ML and hilus.

### Effect of APP/PS1 genotype

**Fig. 6:**
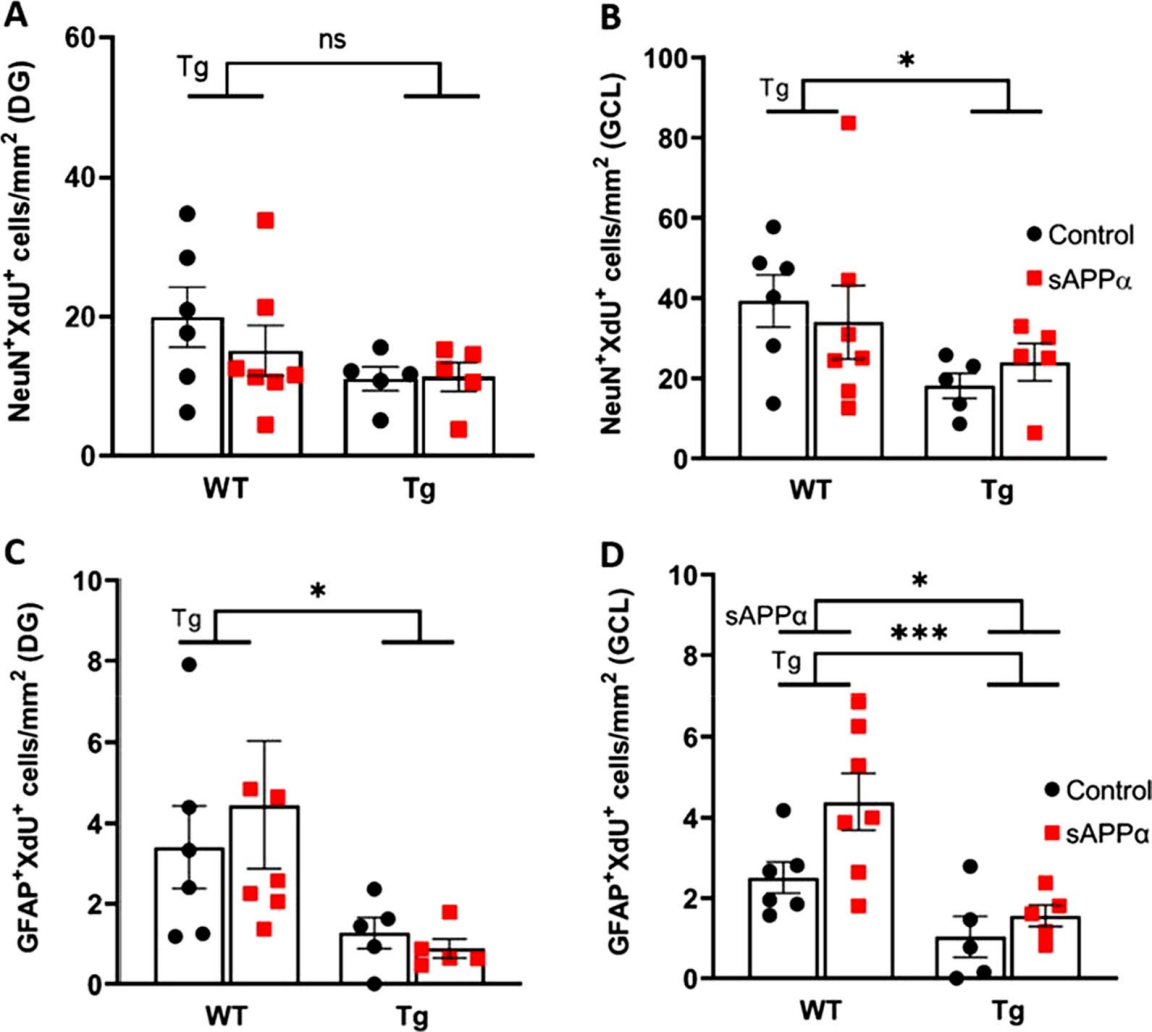
sAPPα effects on cell survival in the DG. **A** Area density measurements of NeuN^+^XdU^+^ cells in the whole dentate gyrus. **B** Area density measurements of NeuN^+^XdU^+^ cells in the granule cells layer only. **C** Area density measurements of GFAP^+^XdU^+^ cells in the whole dentate gyrus. **D** Area density measurements of GFAP^+^XdU^+^ cells in the granule cell layer only. *Tg* main effect of genotype, *sAPPα* main effect of sAPPα treatment as assessed by two-way ANOVA. *ns* not significant; *p < 0.05; ***p < 0.001. Data expressed as mean ± SEM

There was a significantly reduced neuron (NeuN^+^XdU^+^) area density in the GCL (main effect of genotype F_(1, 19)_ = 4.804, *p* = 0.041; Fig. 6B). In the whole DG, however, there was no main effect of genotype (F_(1, 19)_ = 3.392, *p* = 0.467; Fig. 6A). Thus, the genotype effect on adult-born neuronal survival was confined to the GCL. Astrocyte (GFAP^+^XdU^+^) area density was also significantly reduced in the GCL of the Tg mice (main effect of genotype F_(1, 19)_ = 15.63, *p* < 0.001; Fig. 6D). In this case, the effect was also present across the whole DG (F_(1, 19)_ = 6.225, *p* = 0.022; Fig. 6C).

### Effect of sAPPα overexpression

There were no main effects of sAPPα treatment on neuron area density in either the GCL alone (F_(1, 19)_ = 0.001, *p* = 0.972; Fig. 6B) or the whole DG (F_(1, 19)_ = 0.4414, *p* = 0.514; Fig. 6A) and no interaction between genotype and sAPPα treatment in the GCL alone (F_(1, 19)_ = 0.620, *p* = 0.441) or the whole DG (F_(1, 19)_ = 0.620, *p* = 0.441). In contrast, sAPPα treatment caused a significant increase in astrocyte area density in the GCL (F_(1, 19)_ = 4.884,* p* = 0.040; Fig. 6D), although not in the whole DG (F(1, 19) = 0.081, p = 0.779; Fig. 6C). Taken together, these results indicate that the density of both neurons and astrocytes born 8 weeks previously was significantly reduced in the Tg mice, especially in the GCL. The genotype effect on astrocytes was maintained despite the increase caused by sAPPα treatment, indicating that the sAPPα affected both genotypes.

### Differentiation of adult-born cells

**Fig. 7:**
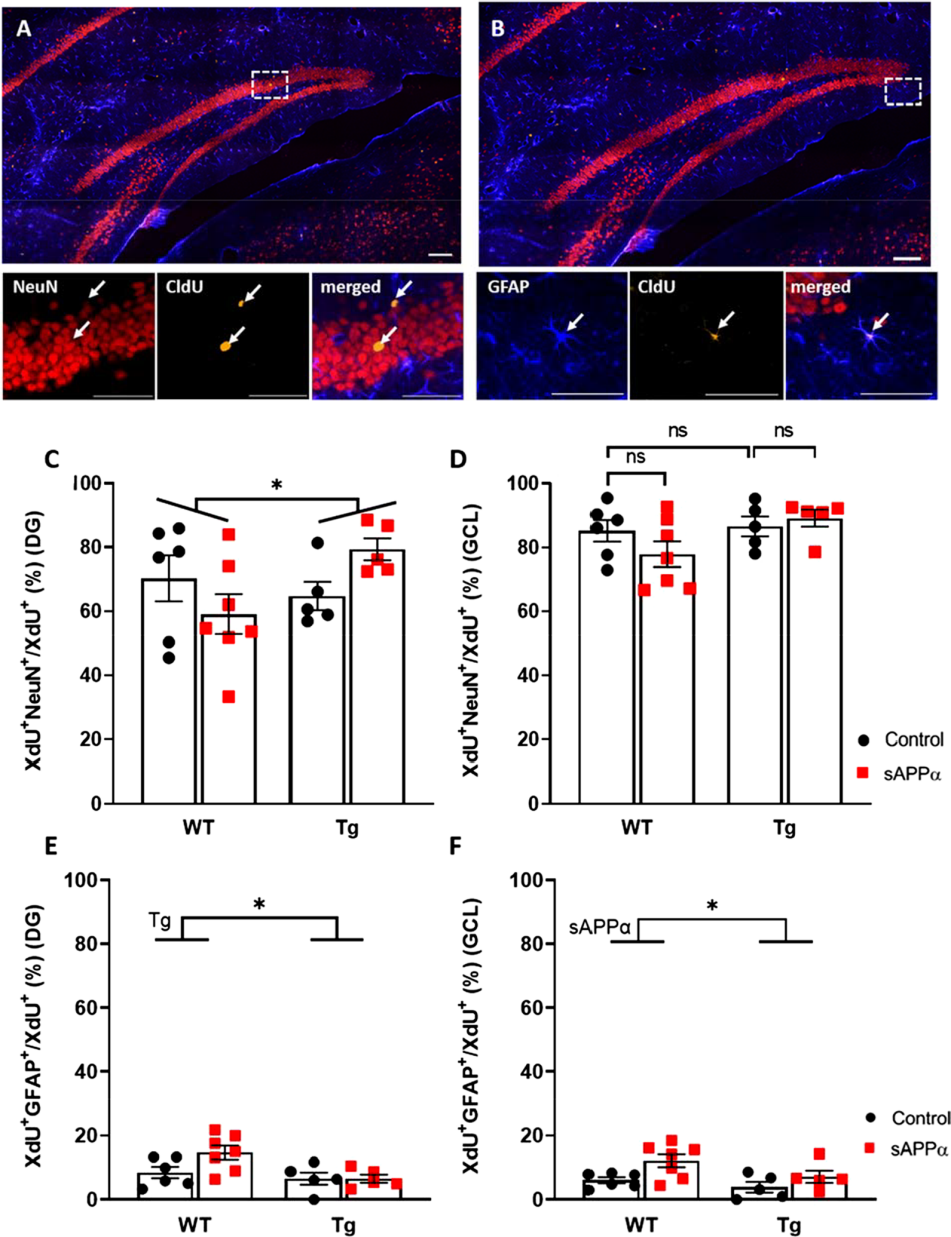
sAPPα effect on cell differentiation. **A** A representative mosaic confocal image of the dentate gyrus. For all images NeuN is red, GFAP is blue and CldU is yellow. Scale bar: 100 µm. **A**, below From the top left insert: NeuN labelling, CldU labelling, and merged image showing two NeuN + CldU + cells. Scale bar: 50 µm. **B** A representative mosaic confocal image of the dentate gyrus. Scale bar: 100 µm. **B**, below From the top right insert: GFAP labelling CldU labelling, and merged image showing two GFAP + CldU + cells. Scale bar: 50 µm. **C** Results of the differentiation assay for neurons in the whole dentate gyrus of WT and Tg animals. There was a significant APPα treatment x genotype interaction, *, p < .05. **D** Results of the differentiation assay for neurons in the granule cell layer of WT and Tg animals. **E** Results of the differentiation assay for astrocytes in the whole dentate gyrus of WT and Tg animals. **F** Results of the differentiation assay for astrocytes in the granule cell layer of WT and Tg animals. Tg, main effect of genotype; sAPPα, main of sAPPα treatment as assessed by 2-way ANOVA. *ns* not significant, *p < .05. Data expressed as mean ± SEM

To determine the effect of genotype and sAPPα expression on neuronal versus astrocytic differentiation, the percentage of either XdU^+^NeuN^+^/XdU^+^ or XdU^+^GFAP^+^/XdU^+^ cells relative to all labelled cells was compared between groups (Fig. 7). Double-labelling with XdU and NeuN revealed that the vast majority of XdU^+^ cells in the DG became neurons in all mice, and even more so in the GCL specifically (Fig. 7D), whereas a small percentage of XdU^+^ cells in the DG became astrocytes (Fig. 7E, F). There were also a small proportion of XdU-labelled cells that could not be identified (data not shown).

### Effect of APP/PS1 genotype

There was no main effect of genotype on the percentage of neurons (XdU^+^NeuN^+^/XdU^+^) in the GCL alone (F_(1, 19)_ = 0.081, *p* = 0.779; Fig. 7) or the whole DG (F_(1, 19)_ = 1.536, *p* = 0.230; Fig. 7C, E). In contrast, there was a strong trend toward a decrease in the percentage of astrocytes (XdU^+^GFAP^+^/XdU^+^) in the GCL in the Tg mice, (F_(1, 19)_ = 4.206, *p* = 0.054; Fig. 7F), and a significant decrease in the whole DG (F_(1, 19)_ = 6.766, *p* = 0.018; Fig. 7E). Together these results indicate that astrocytic differentiation was reduced in the whole DG of Tg mice compared to WT mice irrespective of sAPPα treatment.

### Effect of sAPPα overexpression

There were no main effects of sAPPα treatment on the percentage of neurons in the GCL only (F_(1, 19)_ = 0.448, *p* = 0.511; Fig. 7D) or the whole DG (F_(1, 19)_ = 0.086, *p* = 0.773; Fig. 7C). However, there was a significant interaction between genotype and sAPPα treatment specifically in the whole DG (F_(1, 19)_ = 4.685, *p* = 0.043). Thus, sAPPα expression tended to increase neuronal differentiation in Tg mice but to decrease it in WT mice in the whole DG (Fig. 7C), an effect mostly in areas outside of the GCL. For astrocytes, there was a small but significant increase in relative expression for the sAPPα-treated mice in the GCL alone (F_(1, 19)_ = 7.354, *p* = 0.014; Fig. 7F), but not in the whole DG (F_(1, 19)_ = 2.609, *p* = 0.123; Fig. 7E). Unlike for neuronal differentiation, there was no significant genotype x sAPPα interaction for the percentage of astrocytes in the whole DG (F_(1, 19)_ = 3.151, *p* = 0.092). A summary of the main findings for adult-born neuronal and astrocytic differentiation is presented in Table 1.

**Table 1:** Summary of the main effects of genotype and sAPPα treatment and their interaction on cell differentiation

% Differentiation	Neuronal		Astrocytic	
Dependent variable	Genotype	sAPPα	Genotype	sAPPα
GCL	=	=	=	↑^*^
DG	=	↑/↓^a^	↓^*^	=

↓ denotes reduction; ↑ denotes increase; = denotes no change. ^a^Denotes an interaction between genotype and sAPPα treatment whereby sAPPα tended to increase neuronal differentiation in Tg mice but to decrease it in WT mice. *p < .05. The DG was comprised of the GCL, ML and hilus

### Effect of sAPPα overexpression on amyloid plaque burden

**Fig. 8:**
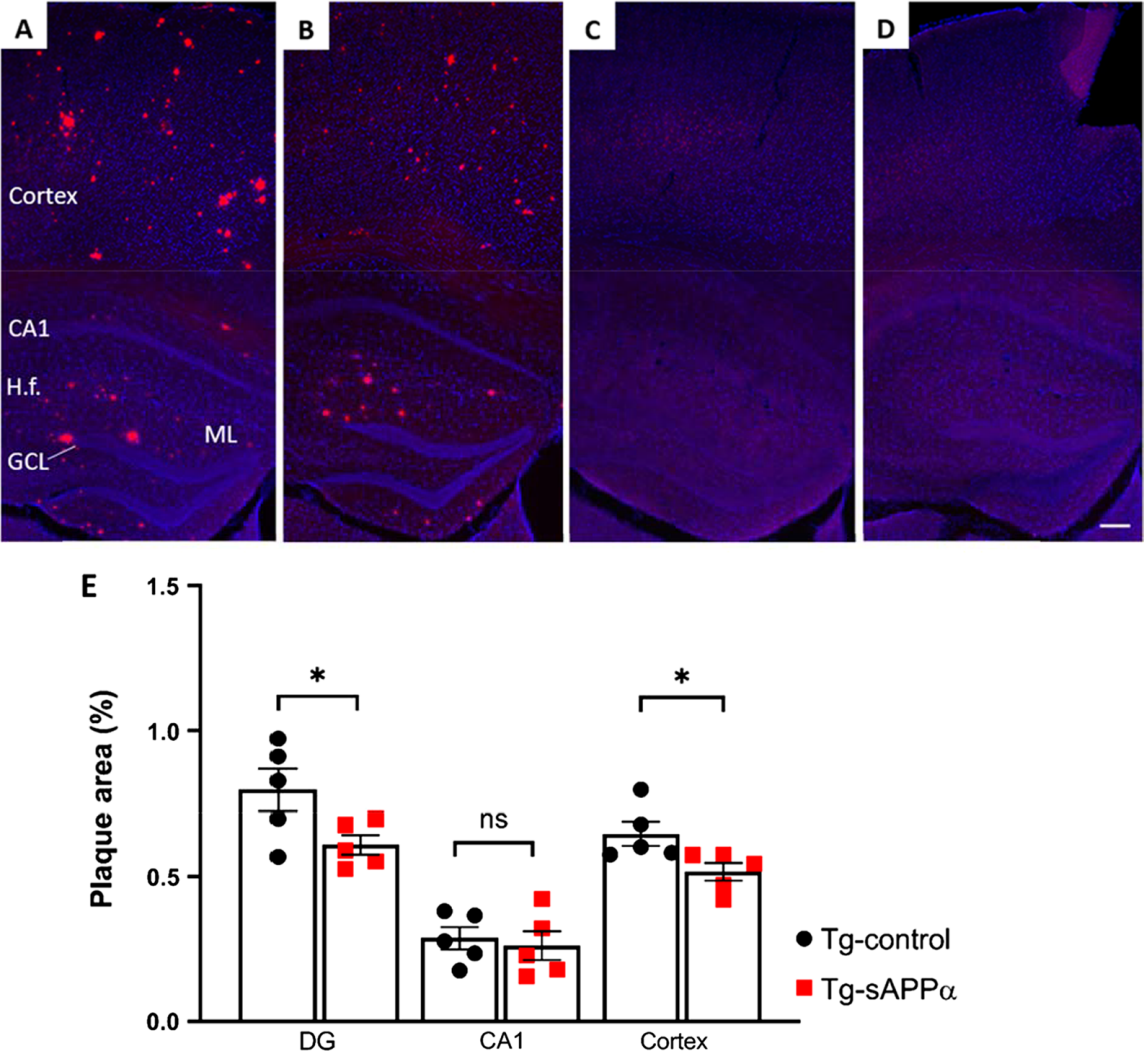
sAPPα effects on amyloid plaque load. Amyloid plaques (Congo red) were specific to Tg-control (**A**) and Tg-sAPPα (**B**) mice at 11.5 months of age in the hippocampus and cortex (DAPI nuclei, blue). No amyloid plaques were present in WT-control (**C**) and WT-sAPPα (**D**) mice the same age. Scale bar: 100 µm. *H.f.* Hippocampal fissure. **E** Percentage of area covered by plaques measured across the dentate gyrus, CA1 and the somatosensory cortex for the Tg-control and Tg-sAPPα overexpressing mice. *ns* not significant, *, p < 0.05 assessed unpaired Student’s *t*-test. Data expressed as mean ± SEM

The amyloid plaque burden was evaluated in the whole DG, CA1, and overlying sensorimotor cortex by Congo red staining. Plaque deposition was more abundant in the ML and hilus compared to the GCL (Fig. 8A, B), which is consistent with our observations in a different APP/PS1 mouse strain [17]. The percentage plaque-covered area was significantly reduced in Tg-sAPPα mice by 24% in the DG (Tg-control vs. Tg-sAPPα: 0.80 ± 0.08% vs. 0.61 ± 0.08%, *t*_(8)_ = 2.317, *p* = 0.049, n = 5 per group) and by 20% in the cortex (Tg-control vs. Tg-sAPPα: 0.65 ± 0.05% vs. 0.52 ± 0.05%, *t*_(8)_ = 2.518, *p* = 0.036, n = 5 per group; Fig. 8E). Few plaques were detected in the CA1 region and the percentage of plaque area in CA1 was not different between Tg-control and Tg-sAPPα mice (Tg-control vs. Tg-sAPPα: 0.29 ± 0.06% vs. 0.26 ± 0.06%, n = 5 per group, *t*_(8)_ = 0.4104, *p* = 0.692; Fig. 8E). No amyloid plaques were detected in the hippocampus or cortex of WT-control (Fig. 8C) and WT-sAPPα mice (Fig. 8D).

## Discussion

This study set out to investigate whether adult hippocampal genesis of neurons and astrocytes was impaired in an APP/PS1 AD mouse model and, if so, whether any effects could be rescued by AAV-mediated sAPPα overexpression. First, it was important to determine the extent to which the AAV9-HA-HA-sAPPα virus transduced cells in the dentate gyrus. All injected AAV9-HA-HA-sAPPα mice exhibited prominent sAPPα and HA expression around the cell bodies located in the GCL and hilus of the DG, indicating high transduction efficiency that was consistent with that found by Fol et al. [18]. AAV9 achieved a greater spread of sAPPα compared to the more restricted spread using a lentiviral vector, as employed by Tan et al. [17]. All AAV-HA-HA-sAPPα injected mice also exhibited similar patterns of sAPPα within the CA1-3 subfields, most notably in proximal CA3 pyramidal cells. AAV-HA-HA-sAPPα spread through the dorsal–ventral extent of the hippocampus, even though the injection site was in the dorsal hippocampus only. Long-term expression of sAPPα was driven by the synapsin promoter, permitting sAPPα to be expressed exclusively in neurons. Although not quantified in the present study, Fol et al. demonstrated with a similar virus (although with two injection sites) that, four weeks after injection, a significant threefold increase in sAPPα expression was found in the virally transduced animals relative to the endogenous sAPPα in WT mice [18]. As an additional advantage, sAPPα is secreted from cells, so its effects on cells could have been throughout the entire region in which it was present and may well have included effects on astrocytes and other cell types within the DG such as neural progenitor cells.

### Rescue of cell proliferation by sAPPα in APP/PS1 mice

Adult hippocampal neurogenesis studies utilising transgenic mouse models of AD have consistently shown that the adult hippocampal cell proliferation is impaired [38–41]. Thus, cell proliferation in the SGZ was hypothesised to be decreased in APP/PS1 mice compared to WT mice in the present study. Consistent with this hypothesis, APP/PS1-control mice displayed a 71% reduced linear density of newly proliferated cells in the SGZ compared to the WT-control mice. This decrease may have been due to a reduction in the number of activated stem cells and therefore, the synthesis of progenitors. Additionally, production of Aβ soluble aggregates may have decreased the rate of cell cycle completion by increasing the cell cycle length. While a small number of studies reporting increased AHN [42, 43] in AD mouse models attributed their findings to differences in genetic backgrounds and transgene expressions and the timing of aberrant AHN in relation to the neuropathology stage, the currently observed cell proliferation deficit at 11.5 months of age is consistent with previous findings of impaired cell proliferation in the SGZ of APP/PS1 mice from as early as 1–2 months [44], by 3 months [45], by 6 months [46] and between 8–9 months of age [47].

We found that sAPPα overexpression significantly increased cell proliferation in the SGZ of the Tg mice by ~ 150% but not in the WT mice, producing a near-full rescue of cell proliferation in the Tg- sAPPα mice. Whether this increase in proliferation in the APP/PS1 mice was due to an expansion of the proliferative pool and synthesis of progenitors, or a faster rate of cell cycle completion could not be distinguished in the present study.

sAPPα has been shown to stimulate the proliferation of adult NPC in the SVZ in normal animals in an epidermal growth factor and basic fibroblast growth factor-dependent [48] or independent manner [49]. The lack of effect in the SGZ in these studies is consistent with our lack of effect in the WT-control animals. Also consistent with this study, sAPPα has been demonstrated to rescue proliferation deficits induced by β-secretase inhibition [49] and ageing [50], indicating that sAPPα can enhance proliferation under impairment conditions, both with reduced Aβ and in the presence of increased Aβ burden. While the effect of sAPPα on the cell division process may be relatively direct, it is noteworthy that endogenous Aβ plays a causal role in impairing proliferation in early AD [51] and that sAPPα can reduce the Aβ plaque burden in the DG (Fig. 8; [18]). Overexpression of sAPPα may thus have reduced Aβ-mediated interference of levels of endogenous sAPPα and other growth factors, leading to improvements in the brain environment [18], including the neurogenic environment that promoted cell proliferation. sAPPα overexpression would have restored and raised sAPPα levels in the local environment, making it more conducive for expansion of the progenitor pool.

The lack of proliferative effects in WT mice may be related to the degree of sAPPα overexpression. In neurospheres from normal rats, sAPPα dose-dependently increased cell proliferation over a range of concentrations (0.01–1 nM) [52]. However, these experimental conditions were different from the present study in whole animals. Given the myriad of known beneficial effects of sAPPα [53], it was intuitive to infer this excess would be promising for AHN in a WT mouse. However, in assays of neurogenesis, neuroprotection and synaptic plasticity, sAPPα confers benefit only at low concentrations [8, 52, 54]. For example, there was an inverted-U shaped dose-dependent rescue of proliferation deficits in adult mouse SVZ-derived neurospheres [49]. Thus, a plausible explanation for the observed lack of effect on SGZ proliferation in WT- sAPPα mice is that sAPPα overexpression was on a background of an already optimal concentration of sAPPα in the tissue.

### Failure of sAPPα to rescue cell survival in APP/PS1 mice

Survival of adult-born cells, in particular neurons and astrocytes, was hypothesised to be decreased in APP/PS1 mice compared to WT mice. Consistent with the hypothesis, 8-week-old adult-born XdU^+^ cell area density was reduced in the DG of APP/PS1 mice compared to WT mice, irrespective of sAPPα treatment. The same effect was also apparent for both neurons and astrocytes as independently assessed. This apparent impairment in adult-born cell survival is consistent with a previous report in the DG of APP/PS1 mice [39]. However, given the reduced cell proliferation in 11.5 months old APP/PS1 mice in the present study, the reduced survival of adult-born cells including neurons and astrocytes in APP/PS1 mice might be at least partially due to a decreased cell proliferation at the time of injection (i.e. 9.5 months). Whether the decline in cell proliferation at 9.5 months of age was as strong as the 71% reduction in cell proliferation in Tg-control mice that we measured at 11.5 months of age is not known. But since the total cell survival declined by less than 50% in the whole DG, a prediction from this study is that proliferation was not as badly affected at the earlier age of 9.5 months, unless there was some compensatory increase in cell survival mechanisms that offset a more extensive decline in cell genesis. As a future study, DCX^+^/XDU^+^ or NeuN^+^/XdU^+^ cells could also be examined 3–4 weeks post-injection, as this is a time when the adult-born cells play a particularly important role in certain memory tasks. After this up to 50% of cells do not survive past 3–4 weeks post-birth due to apoptosis, which may influence our analysis of survival rates at 8 weeks.

Despite its rescue of cell proliferation in the Tg mice, sAPPα overexpression failed to rescue the survival of XdU^+^-labelled cells or neurons specifically but, interestingly, there was a significant increase in the area density of astrocytes in the GCL across both genotypes. Whether this is due to a protection of astrocyte survival post-genesis by sAPPα, as suggested in a neurosphere study [52], or a delayed migration of astrocytes outside of the SGZ/GCL area remains to be determined.

### Neuronal versus astrocytic differentiation

We also investigated whether the percentage of cells differentiating into neurons or astrocytes showed genotype or treatment effects. Overall, there was no change in the percentage of 8-week-old adult-born neurons (XdU^+^NeuN^+^/XdU^+^) in the GCL or the whole DG of APP/PS1 and WT mice irrespective of sAPPα treatment. In contrast, previous studies demonstrated impaired neuronal differentiation in APP/PS1 mice, as indicated by a reduced proportion of BrdU^+^NeuN^+^/BrdU^+^ cells in the 6 mo old DG [39] and reduced number of DCX^+^/BrdU^+^ cells in the 9 mo old SGZ [44]. This result however is consistent another study of these mice at 10 months of age for which there was no change in the total number of DCX + cells[55], and consistent with studies in other AD mouse models showing no effect on neuronal differentiation [41, 56]. A possible explanation for these inconsistencies might be variations in Aβ pathology within the AD animal models.

sAPPα overexpression was hypothesised to enhance neuronal and/or astrocytic differentiation in sAPPα-treated mice, based on in vitro observations that sAPPα can promote neuronal differentiation [57, 58] and astrocytic differentiation [52] of NPCs. It is notable then that sAPPα overexpression modestly increased astrocytic differentiation, as indicated by the percentage of adult-born astrocytes in the GCL irrespective of genotype, supporting the notion that within the progenitor cell pool, sAPPα promoted astrocyte-committed progenitors to terminally differentiate. Notably, recent studies have emphasised the importance of astrocytes to learning and memory in the hippocampal formation [59], and thus sAPPα overexpression resulting in increased astrocytic differentiation might have contributed to memory enhancement seen in previous studies [17, 18]. In contrast, neuronal differentiation was mainly unchanged in the GCL of both genotypes, consistent with a previous finding that sAPPα treatment did not affect the number of neurons (MAP2^+^ or calbindin^+^) generated from SGZ-derived WT NPCs in vitro [52]. However, in the whole DG, there was a significant interaction between genotype x sAPPα treatment, suggesting that sAPPα overexpression tended to increase neuronal differentiation in APP/PS1 mice but to decrease it in WT mice.

Over 70% of XdU^+^ cells were identified as neuronal cells in the whole DG of the WT-control animals and over 80% in the GCL, roughly in accord with other rodent [60, 61] studies which report ~ 80%. Eight percent of cells were identified as astrocytes at eight weeks in the DG of WT-control mice, similar to rodent studies reporting 7% [62, 63]. This left an average of 22% of XdU^+^ cells expressing neither NeuN nor GFAP in the whole DG across groups, also consistent with previous reports of 20% [60, 64, 65]. Recent studies suggest that unidentified XdU^+^ cells could be microglia, which can arise from progenitor cells [66]. The unidentified XdU^+^ cells in this study were included in the total number of XdU^+^ cells used for differentiation analysis and thus influenced overall neuronal and astrocyte proportions. Future studies are needed to include co-staining with markers of oligodendrocytes and microglia alongside assessing morphology to at least preclude these glial cell types. Another possibility is that these XdU^+^ cells are potential neurons or astrocytes, remaining undifferentiated until their function is determined at a later time [67, 68]. However, as no differences were found in the area density of unidentified XdU^+^ cells in the GCL between any groups, the distribution of these cells did not contribute significantly to the present results.

### Amelioration of Aβ plaque load

As expected, a significant Aβ plaque load was observed in the hippocampus and cortex of the 11.5 mo old APP/PS1 mice compared to WT mice, where no deposits were found. sAPPα’s anti-amyloidogenic properties via direct and indirect inhibition of β-secretase have previously been demonstrated [18, 69, 70]. Consistent with these findings, sAPPα overexpression resulted in a significant reduction in Aβ plaque load in the DG and cortex, indicating that sAPPα overexpression reduced not only Aβ plaque area in the treated hippocampus but also adjacent cortical areas. These findings are in agreement with Fol et al., who reported a significantly reduced plaque area in the cortex and hippocampus as well as a comparable 33% reduction of human Aβ42 species in these areas [18]. This result and the present one can be attributed to efficient diffusion of small AAV-HA-HA-sAPPα particles throughout the hippocampus as well as from the needle tract to the overlying cortex [71]. Another possibility is that secreted sAPPα diffused through the brain parenchyma [18]. There was no significant change in plaque area in the CA1, most likely a result of the small number of plaques in both Tg-control and Tg-sAPPα mice in this region.

A potential mechanism by which sAPPα attenuated the plaque burden was by microglia recruitment, as indicated by the previously reported increased expression of plaque-associated microglia and expression of Triggering Receptor Expressed on Myeloid’ cells (TREM), and of the microglial proteases neprilysin and insulin-degrading enzyme in AAV-HA-HA-sAPPα mice [18]. Although not tested in the present experiments, these prior data suggest that in the present study sAPPα may have helped recruit microglia into the vicinity of amyloid plaques, potentially enhancing Aβ and plaque clearance (Additional file 1).

## Conclusion

This is the first study to assess the effect of in vivo sAPPα overexpression on adult hippocampal neuro-and astrogenesis in an AD mouse model. We observed a rescue of the APP/PS1 deficit in proliferation of adult born cells, an increase in astrocytic differentiation in the GCL of APP/PS1 mice, and the survival of more astrocytes in both WT and APP/PS1 mice after overexpression of sAPPα. Finally, we also found a decrease in amyloid plaque levels after treatment. Our results may reveal one of the cellular mechanisms for memory rescue found with sAPPα overexpression in previous studies [17, 18]. These findings, together with the known importance of adult-hippocampal neurogenesis in memory formation and retention, support the possibility that sAPPα could be used therapeutically in AD to restore memory [53].

## Supplementary Information

**Additional file 1.** Excel file holding the raw data for the results reported in this paper.

## Data Availability

Data and materials will be made upon reasonable request to the corresponding author.
